# Role of mTOR inhibitor in cholangiocarcinoma cell progression

**DOI:** 10.3892/ol.2014.1799

**Published:** 2014-01-15

**Authors:** PENPAK MOOLTHIYA, RUTAIWAN TOHTONG, SIRIPORN KEERATICHAMROEN, KAWIN LEELAWAT

**Affiliations:** 1 Department of Biochemistry, Faculty of Science, Mahidol University, Rajathevi, Bangkok 10400, Thailand; 2 Department of Molecular Medicine, Faculty of Science, Mahidol University, Rajathevi, Bangkok 10400, Thailand; 3 Department of Surgery, Rajavithi Hospital, Rajathevi, Bangkok 10400, Thailand

**Keywords:** mTOR inhibitor, RAD001, cholangiocarcinoma

## Abstract

Cholangiocarcinoma (CCA) is a lethal malignancy of the biliary epithelium. CCA is resistant to currently available chemotherapy; therefore, new drugs as well as new molecular targets must be identified for the development of an effective treatment for CCA. The present study showed that RAD001 (everolimus), a derivative of rapamycin and an orally bioavailable mammalian target of rapamycin (mTOR) inhibitor, exhibits cytotoxic and antimetastatic effects in a CCA cell line, RMCCA-1. Treatment with low concentrations of RAD001 resulted in a significant reduction of *in vitro* invasion and migration of RMCCA-1, concomitant with a reduction of filopodia and alteration of the actin cytoskeleton. Although, matrix metalloproteinase-9 and -14 activities were unaltered. However, at high concentrations, RAD001 exhibited cytotoxic effects, reducing cell proliferation and inducing apoptotic cell death. Overall, RAD001 exhibits multiple effects mediated by the inhibition of the mTOR, which may serve as a promising agent for the treatment of CCA.

## Introduction

Cholangiocarcinoma (CCA) is a highly malignant epithelial neoplasm that arises from the biliary epithelium. It is a major public health issue in Northeastern Thailand. CCA is highly lethal due to lack of early detection, resistance to chemotherapy and high propensity to invade locally and distantly (1). Currently, no effective therapy has been identified for the disease and alternative therapeutic options are urgently required.

Increased effort in recent years has been invested into identifying new cellular targets important for cancer cell survival and metastasis. Mammalian target of rapamycin (mTOR), a 289-kDa serine/threonine kinase and a downstream effector of the phosphoinositide 3-kinase (PI3K)/Akt signaling pathway, was identified as a promising therapeutic target for the treatment of a number of types of cancer, including CCA (2–4). mTOR forms the following two distinct multiprotein complexes:Rapamycin-sensitive mTOR complex 1 (mTORC1); and rapamycin-insensitive complex 2 (mTORC2). mTORC1, consisting of raptor and G protein β subunit-like, regulates a number of cellular processes, including cell proliferation, differentiation and cell cycle progression by phosphorylating the ribosomal protein S6 kinase (S6K) to stimulate protein translation and ribosome biogenesis (5). Activation of mTORC1 also leads to the phosphorylation and inactivation of the eukaryotic initiation factor 4E binding protein-1 (BP1), promoting the protein translation (6). Rapamycin (sirolimus), the first identified mTOR inhibitor, is extracted from the bacterial strain *Streptomyces hygroscopicus* found in soil, and has been used as an antifungal agent (7). Previous *in vitro* and *in vivo* studies have shown that rapamycin and its analogs exhibit substantial antitumor activity in a number of types of cancer (8–10).

Binding of the rapamycin/FK506-binding protein 12 complex to mTOR promotes the dissociation of the scaffold protein, raptor, from mTORC1, suppressing the mTORC1 function (11). Previous studies have demonstrated that the treatment of hepatocarcinoma (12) and CCA (13) with rapamycin significantly inhibits cell growth and induces temporary partial remission or stable disease. Previously, an orally bioavailable rapamycin derivative, RAD001 [40-O-(2-hydroxyethyl)-rapamycin or everolimus; Novartis International AG, Basel, Switzerland], was developed with the aim of targeting the mTOR pathway. In 2009, RAD001 was approved by the Food and Drug Administration for the treatment of advanced renal cell carcinoma (14). However, whether RAD001 is effective against CCA is unknown.

The present study investigated the effects of RAD001 on the malignant phenotypes of CCA *in vitro*, using the RMCCA-1 cell line as a model. It was demonstrated that RAD001 suppresses *in vitro* invasiveness and alters the actin cytoskeleton at low, non-toxic concentrations, concomitant with a significant reduction of p-mTOR and p-extracellular signal-regulated kinase (ERK)1/2 levels. At high concentrations, RAD001 exhibited cytotoxic effects, reducing cell proliferation and inducing apoptosis. Overall, the results of the current study demonstrated that RAD001 exhibits multiple effects on the CCA cells, suggesting that it may serve as a potential therapeutic agent for the treatment of CCA.

## Materials and methods

### Cell cultures

The RMCCA-1 human intrahepatic CCA cell line (15) was grown in HAM’s F12 medium (GIBCO, Grand Island, NY, USA) supplemented with 10% fetal bovine serum (FBS) at 37°C in a 5% CO_2_ humidified atmosphere.

### Cell proliferation assay

RMCCA-1 cells were seeded in 96-well culture plates at a density of 10,000 cells per well overnight prior to the addition of various concentrations of RAD001 (0–10 μM). After 24 and 48 h, water-soluble tetrazolium salts 1 (WST-1) cell proliferation assay reagent (Roche Diagnostics, Laval, QC, Canada) was added to the culture media and incubated for an additional 2 h before the optical density (OD) at 450 nm was read. The percentage of proliferation was calculated based on the number of control [dimethyl sulfoxide (DMSO)-treated] cells. Three independent experiments were performed, in triplicate.

### Detection of apoptosis by terminal deoxynucleotidyl transferase-mediated dUTP nick end labeling (TUNEL) assay

RMCCA-1 cells were grown on sterile coverslips and allowed to attach for 24 h prior to being treated with 0, 0.5 and 2 μM RAD001 for 24 h. The number of apoptotic cells was determined using the Apo-BrdU TUNEL assay kit (Invitrogen Life Technologies, Carlsbad, CA, USA) according to the manufacturer’s instructions. Briefly, cells were washed with cold phosphate-buffered saline (PBS; pH 7.4) and fixed with 1% paraformaldehyde and ice-cold 70% ethanol for 30 min. Fixed cells were then labeled with BrdUTP using terminal deoxynucleotide transferase in a humidified chamber at 37°C for 1 h, followed by staining with Alexa Fluor 488-conjugated anti-BrdU antibody for 30 min at room temperature. The number of apoptotic cells was quantified by counting the number of cells with green fluorescent dots. Total cell number was determined by counting the nuclei stained with DAPI in 10 fields under a fluorescence microscope (magnification, ×20; (Olympus IX71, Olympus Corporation, Tokyo, Japan). The degree of apoptosis was presented as the percentage of apoptotic cells compared with the total number of cells.

### In vitro invasion and migration assay

The *in vitro* invasion of CCA cells was assayed using a Transwell chamber with 8-μm pore inserts (24-well cell culture; Costar, Boston, MA, USA) that were coated with 100 μl Matrigel (0.3 mg/ml; Becton-Dickinson, Bedford, MA, USA). RMCCA-1 cells were pretreated with 0, 0.1 and 0.5 μM of RAD001 for 6 h in culture media with 1% FBS at 37°C prior to the harvesting of the cells. In total, 5×10^4^ cells were seeded into the upper chamber of the Transwell containing serum-free media, whereas media with 1% FBS was added into the lower chamber as a chemoattractant. RAD001 was added to the two chambers. Following incubation for 18 h, the filters were fixed and stained with hematoxylin. The number of invaded cells per well was counted in 10 fields under the microscope (magnification, ×10) and presented as a percentage of control (DMSO-treated) cells. *In vitro* migration assays were also performed using the Transwell chambers in a similar manner to the *in vitro* invasion assay, with the exception that no Matrigel coating was applied on the filters.

### Detection of actin polymerization

RMCCA-1 cells were grown on sterile coverslips and allowed to attach overnight prior to being treated with RAD001 or DMSO for an additional 24 h. The cells were washed twice with PBS, fixed with 4% paraformaldehyde and permeabilized in 1% Triton X-100 for 15 min. Non-specific binding was blocked with 1% bovine serum albumin prior to probing the cells with Alexa Fluor 488-conjugated phalloidin (Molecular Probes, Eugene, OR, Canada) for 30 min. Following washing with PBS, coverslips were mounted on the slide glass containing ProLong^®^ Gold Antifade reagent (Invitrogen Life Technologies) and examined under a confocal microscope (magnification, ×60; Olympus SV1000, Olympus Corporation) using Olympus FV10-ASW 1.7 software (Olympus Corporation).

### Gelatin zymography assay

RMCCA-1 cells were starved for 24 h in serum-free media containing RAD001 or DMSO prior to the collection of the conditioned medium. The conditioned medium was then mixed with non-reducing sample buffer [2% SDS, 10% glycerol, 62.5 mM Tris-Cl (pH 6.8) and 0.01% bromophenol blue] and separated on a 7.5% polyacrylamide gel containing gelatin (1 mg/ml). Following electrophoresis, the gel was soaked in two changes of 2.5% Triton X-100 for 30 min followed by an incubation in 50 mM Tris-Cl (pH 7.5), 10 mM CaCl_2_, 1 μM ZnCl_2_ and 1% Triton X-100 for 18 h at 37°C. The gel was then stained with 0.25% (w/v) Coomassie blue R250 for 2 h. The gelatinolytic activity was observed as a clear band on the blue background of the Coomassie-stained gel and documented using a Bio-Rad GS700 gel scanner (Bio-Rad, Hercules, CA, USA).

### Western blot analysis

RMCCA-1 cells (~5×10^5^) were seeded in a 100-mm culture plate overnight prior to the addition of various concentrations of RAD001. DMSO-treated cells were used as a control. Following incubation, cells were collected, washed with PBS and lysed in lysis buffer. Next, protein samples were mixed with SDS sample buffer and β-mercaptoethanol, boiled and separated using 7.5% SDS-PAGE. The gel was run for 1.5 h at 180 V prior to transferring the proteins onto a nitrocellulose membrane (Bio-Rad, Hercules, CA, USA) by electroblotting for 2.5 h at 120 V and 4°C. Non-specific binding was blocked using 5% skimmed milk for 1 h at room temperature. The blot was probed with rabbit anti-phospho-mTOR (Ser 2448) antibody and rabbit anti-cleaved caspase 7 antibody, prior to being stripped and reprobed with rabbit anti-mTOR and rabbit anti-caspase 7 antibody, respectively. The antibodies were purchased from Cell Signaling Technology (Beverly, MA, USA). Quantification of matrix metalloproteinase (MMP)-14 was determined by using specific antibody against MMP-14 (Abcam, Cambridge, MA, USA), whereas, β-actin (Cell Signaling Technology) was used as a loading control. Anti-rabbit IgG secondary antibody conjugated with horseradish peroxidase (HRP; Cell Signaling Technology) was used as a secondary antibody. The immunoreactive bands were detected using enhanced chemiluminescence (Amersham Pharmacia Biotech, Amersham, UK) and visualized by exposure to X-ray film.

### Detection of intracellular signaling

The PathScan^®^ intracellular signaling array kit (Cell Signaling Technology) was used, according to the manufacturer’s instructions, to simultaneously detect 18 significant and well-characterized signaling molecules, when phosphorylated or cleaved. Briefly, cells were washed with ice-cold PBS and lysed in cell lysis buffer. The array blocking buffer was added to each well and incubated for 15 min at room temperature. Then, the lysate was added to each well and incubated for 2 h at room temperature. Following washing, the detection antibody cocktail was added to each well and incubated for 1 h at room temperature. The HRP-linked streptavidin was added to each well and incubated for 30 min at room temperature. The slide was then covered with LumiGLO/Peroxide reagent (Cell Signaling Technology) and exposed to film for 2–30 sec.

### Statistical analysis

All experiments were performed in triplicate and data are presented as the mean ± standard error of the mean. Statistical comparisons were performed using Student’s t-test. P<0.05 was considered to indicate a statistically significant difference.

## Results

### Effect of RAD001 on mTOR phosphorylation

**Figure 1:**
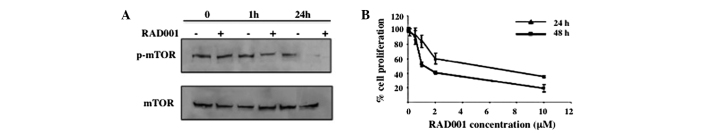
Effect of RAD001 on mTOR phosphorylation and cell proliferation. (A) Suppression of mTOR phosphorylation by RAD001 was determined by western blot analysis. RMCCA-1 cells were treated with 0.1 μM RAD001 for 0, 1 and 24 h, and cells treated with DMSO were used as negative control. Phosphorylation of mTOR was determined using an antibody specific for phosphorylated mTOR at Ser 2448, the Rheb phosphorylation site. Total mTOR was used as a loading control. A representative of three independent experiments is shown. (B) Effect of RAD001 on cell proliferation. RMCCA-1 was treated with 0, 0.1, 0.5, 1, 2 and 10 μM RAD001 for 24 and 48 h, prior to the assessment of cell proliferation by water-soluble tetrazolium salts 1 assay. The absorbance at 450 nm was measured. Data are presented as the mean ± SEM of the results presented as a percentage of the control (DMSO-treated) cells from three independent experiments, each performed in triplicate. mTOR, mammalian target of rapamycin; DMSO, dimethyl sulfoxide.

To determine whether RAD001 affects the phosphorylation of mTOR, an antibody specific for phosphorylated Ser 2448, a Rheb-catalyzed phosphorylation site that has been shown to associate predominantly, but not exclusively, with mTORC1, was used (16). As shown in Fig. 1A, treatment with 0.1 μM RAD001 for 1 h significantly reduced p-mTOR, whereas treatment for 24 h suppressed the phosphorylation of mTOR almost completely.

### Effect of RAD001 on cell proliferation

To identify whether RAD001 has an effect on the cell proliferation of RMCCA-1 cells, a WST-1 assay was performed. RMCCA-1 cells were treated with 0–10 μM RAD001 or DMSO for 24 and 48 h, respectively, prior to being subjected to the WST-1 assay. The OD at 450 nm was measured and was presented as a percentage of the control (DMSO-treated) cells. Treatment with RAD001 resulted in a dose-dependent inhibition of cell proliferation compared with the control at the two time points. A 50% reduction of cell proliferation was achieved at ~5.2 and ~1.3 μM RAD001 at 24 and 48 h, respectively (Fig. 1B).

### Effect of RAD001 on apoptosis

**Figure 2:**
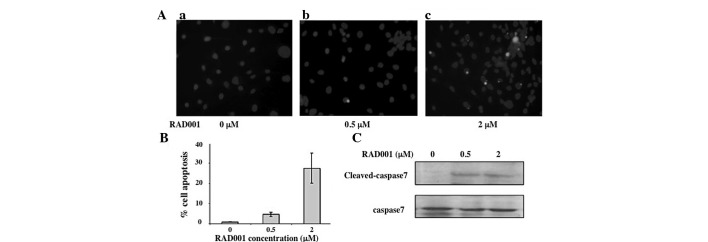
Induction of apoptosis by RAD001 treatment. (A) Images captured from fluorescence microscopy are presented. RMCCA-1 cells were grown on coverslips prior to being treated with various concentrations of RAD001 for 24 h. The degree of apoptosis was determined by TUNEL assay and assessed by counting the number of cells with green fluorescent dots in 10 fields under the fluorescence microscope. Total cell number was determined by staining the nuclei with DAPI. (a) 0, (b) 0.05 and (c) 2 μM RAD001 (magnification, ×20). (B) The degree of apoptosis determined by TUNEL assay was plotted onto a graph and presented as a percentage of the total cell number. Y-axis presents the mean ± SEM of results expressed as a percentage of apoptosis from three independent experiments, each performed in duplicate. Controls (0 μM RAD001) were cells treated with DMSO. X-axis presents the various RAD001 concentrations. (C) Activation of caspase 7 activity was determined by western blot analysis. RMCCA-1 cells were treated with 0.5 and 2 μM RAD001 for 24 h prior to the preparation of cell lysates and subjected to a 7.5% SDS-PAGE. DMSO-treated cells were used as a negative control. The cleaved (active) form of caspase 7 was presented as a 20-kDa band, whereas the full-length (inactive) form was presented as a 35-kDa band. TUNEL, terminal deoxynucleotidyl transferase-mediated dUTP nick end labeling; DMSO, dimethyl sulfoxide.

Apoptosis, a programed cell death, has been shown to be important in the maintenance of the cell population. Impairment of apoptosis checkpoints has been shown to correlate with cancer progression (17). Since the results of the current study demonstrated that treatment with RAD001 results in a reduction of RMCCA-1 cell proliferation, it was further determined if this reduction is associated with the induction of apoptosis using TUNEL assay. RMCCA-1 cells were cultured on coverslips in the presence of various concentrations of RAD001 for 24 h prior to subjecting the cells to TUNEL assay. Cells treated with DMSO (the diluent of RAD001) were used as negative control. Treatment of the RMCCA-1 cells with RAD001 resulted in an induction of apoptosis as shown by the bright green fluorescent dots (Fig. 2A). The proportion of apoptotic cells increased from 4.2±0.9 to 27.5±7.4% with the increasing concentrations of RAD001 from 0.5 to 2 μM (Fig. 2B), respectively.

Apoptosis may be triggered by various extracellular or intracellular stimuli, inducing the extrinsic and intrinsic death signaling pathways, respectively. These diverse death signals, however, eventually activate a common set of executioner caspases, including caspase 3, 6 and 7, leading to apoptotic cell death (18). Caspase 7 activities were assessed by western blot analysis to identify whether they are affected by RAD001 using an antibody against the cleaved (activated) form of caspase 7 (20 kDa). The amount of caspase 7 in each sample was normalized using an antibody against the full length (inactive) caspase 7 (35 kDa). The results showed that a faint band of 20 kDa, representing the cleaved, active form of caspase 7, was present in the DMSO-treated cells. However, the intensity of this band was markedly increased compared with the 35-kDa band identified in cells treated with 0.5 and 2 M RAD001 (Fig. 2C).

### Effect of RAD001 on invasion and migration

**Figure 3:**
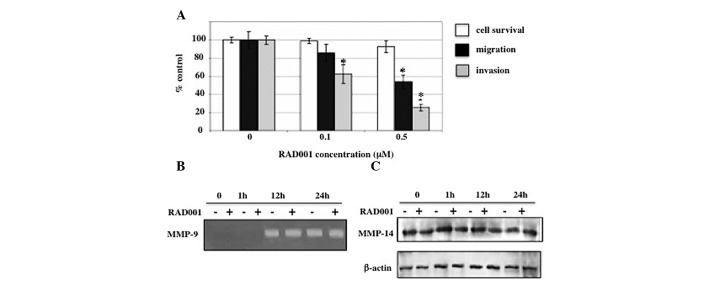
Effects of RAD001 on cell invasion, migration and MMPs. (A) RMCCA-1 cells were exposed to 0, 0.1 and 0.5 μM RAD001 for a total of 24 h in each assay. For the *in vitro* invasion and migration assay, RMCCA-1 cells were pretreated with RAD001 for 6 h prior to being incubated in the Transwell chamber for an additional 18 h. RAD001 was present in the upper and lower chambers. Y-axis presents the mean ± SEM of results expressed as a percentage of the control from three independent experiments, each performed in duplicate. Controls were DMSO-treated cells. X-axis presents the various RAD001 concentrations. (B) Effects of RAD001 on the secretion of MMP-9 into the conditioned medium from RMCCA-1 cells. RMCCA-1 cells were treated with 0.5 μM RAD001 or DMSO (negative control) in serum-free media for 0, 1, 12 and 24 h prior to determining the MMP-9 activity by gelatin zymography. An intense band at 92 kDa corresponding to MMP-9 activity was detected. Equal volumes of conditioned media were loaded into each lane. (C) Effects of RAD001 on MMP-14 protein. RMCCA-1 cells were treated with 0.5 μM RAD001 in serum-free media for 0, 15 min, 1 and 24 h, prior to the preparation of the whole cell lysates, and subjected to 7.5% SDS-PAGE. The blot was then probed with a monoclonal antibody against MMP-14 and β-actin was used as a loading control. ^*^P<0.05, vs. controls. MMPs, matrix metalloproteinases; DMSO, DMSO, dimethyl sulfoxide.

Metastasis is a process by which cancer cells spread from the primary tumor to distant locations. This process depends on the ability of the cancer cells to migrate and invade the surrounding tissues (19). Although a number of previous studies have demonstrated the suppressive effect of RAD001 on cancer growth *in vitro*, few studies have analyzed the effect of RAD001 on its ability to induce the invasion and migration of cancer cells. The effect of RAD001 on *in vitro* invasion and migration was determined by exposing RMCCA-1 cells to 0.1 and 0.5 μM RAD001 for a total of 24 h, at which cell proliferation was inhibited by <10% (Fig. 1B). RMCCA-1 cells were pretreated with RAD001 for 6 h prior to being allowed to invade through the Matrigel-coated Transwell for an additional 18 h in the presence of RAD001. Cell migration assay was performed in a similar manner to the *in vitro* invasion assay, with the exception that Matrigel was not applied to the upper surface of the Transwell filters. Treatment with 0.1 and 0.5 μM RAD001 resulted in a reduction of *in vitro* invasion to 62.6±9.6 and 25.7±3.7%, respectively, compared with the negative control (DMSO-treated) cells. Consistent with the *in vitro* invasion, the presence of 0.1 and 0.5 μM RAD001 reduced the number of migrating cells to 85.9±9.0 and 53.9±7.4%, respectively, compared with the negative control (Fig. 3A).

### Effect of RAD001 on MMPs

Degradation of the extracellular matrix is a key factor which contributes to invasion and metastasis. Since the results showed that RAD001 significantly reduced the *in vitro* invasion and migration of RMCCA-1, the suppression of these processes was analyzed to identify whether it is due to the effect of RAD001 on the secretion of MMPs. Gelatin zymography revealed that the intensity of the 92-kDa band representing the MMP-9 activity was indifferent between RMCCA-1 cells treated with 0.5 μM RAD001 and untreated RMCCA-1 cells, following 12 or 24 h of incubation (Fig. 3B).

In addition to secreted MMPs, membrane-bound MMPs have been previously shown to be crucial in cancer invasion and metastasis. Membrane-type 1 MMP (MT1-MMP or MMP-14), which is expressed on the cell membrane, promotes the invasion of cancer cells by directly degrading extracellular matrix components, including fibronectin, vitronectin, laminin-1 and -5, fibrin, proteoglycans and collagen types I, II and III (20–22). The present study investigated the effect of RAD001 on MMP-14 protein expression using western blot analysis probed with a monoclonal antibody against MMP-14. Consistent with the effect of RAD001 on MMP-9, MMP-14 levels were not affected by the treatment with RAD001 (Fig. 3C).

### Effect of RAD001 on the actin cytoskeleton

**Figure 4:**
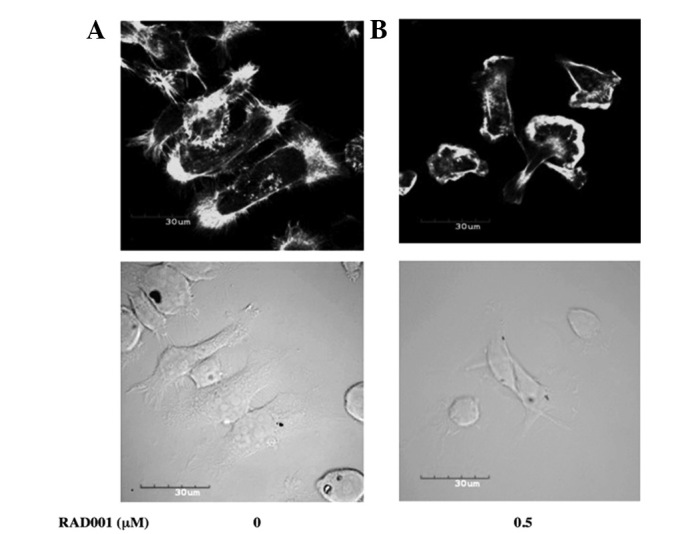
Effects of RAD001 on the filamentous actin cytoskeleton of RMCCA-1 cells. The RMCCA-1 cells were grown on coverslips prior to being treated with RAD001 for 24 h. The actin cytoskeleton was probed with Alexa Fluor 488-conjugated phalloidin and visualized under a confocal microscope. (A) 0 and (B) 0.5 μM RAD001 (magnification, ×60).

The results showed that RAD001 significantly reduced the *in vitro* invasion and migration of the RMCCA-1 cells, suggesting that the actin cytoskeleton may have been affected by RAD001. RAD001 was examined to identify whether it alters the actin cytoskeleton of RMCCA1 by staining actin with Alexa Fluor 488-conjugated phalloidin and by confocal microscopy. RMCCA-1 cells treated with DMSO (negative control) exhibited a well-spread cell morphology with high levels of actin polymerization in the periphery of the cells. Distinct filopodia and lamellipodia were evident. Treatment with 0.5 μM RAD001 for 24 h caused the cells to round up and appear smaller. The lamellipodia and filopodia formation was also diminished (Fig. 4).

### Effects of RAD001 on CCA cell signaling

**Figure 5:**
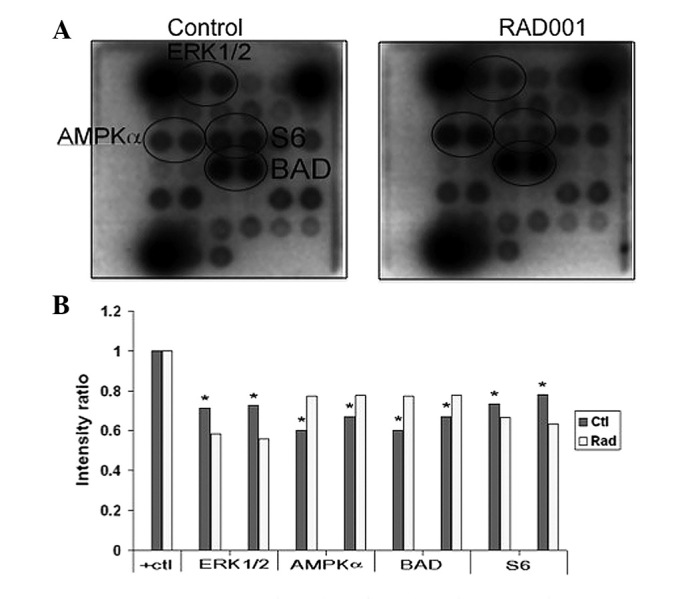
Effects of RAD001 on cholangiocarcinoma cell signaling. (A) Chemiluminescent array images of the PathScan^®^ intracellular signaling array kit revealed various phosphorylated signaling nodes. RAD001-treated cells decreased the phosphorylation of ERK1/2 at Thr202/Tyr204, but increased the phosphorylation of AMPKα at Thr172. Images were captured following brief exposure of the slide to standard chemiluminescent film. (B) The pixel intensity ratio of the phosphorylated signaling molecules. ^*^P<0.05, vs. RAD001. ERK, extracellular signal-regulated kinase; AMPKα; AMP-activated protein kinase α; BAD, Bcl-2-associated death promoter.

To elucidate the signal mediated by RAD001 in CCA cells, the phosphorylation of 18 significant and well-characterized signaling molecules were examined simultaneously using the PathScan intracellular signaling array kit. The results showed that RAD001-treated cells demonstrate a lower extent of phosphorylation of multiple signaling molecules, including ERK1/2, AMP-activated protein kinase α (AMPKα), proline-rich Akt substrate of 40 kDa, Bcl-2-associated death promoter (BAD) and p38, than the control cells (Fig. 5).

## Discussion

To date, despite an improved understanding of CCA pathophysiology, only marginal improvements in the treatment of this disease have been suggested. The PI3K/Akt/mTOR pathway has been shown to be upregulated in CCA cells and this pathway may be a suitable target for the effective treatment of this cancer. RAD001 has been previously shown to inhibit mTOR activity, thereby halting the proliferation of cancer cells, *in vitro* and *in vivo* (23,24). The results of the present study showed that treatment of CCA cells with a high concentration of RAD001 (2 μM) significantly reduced cell proliferation, whereas a low concentration of RAD001 (0.5 μM) impaired cell invasion and the organization of the actin cytoskeleton. These observations are consistent with a previous study, where low concentrations of RAD001 imparted significant effects on cell invasion and migration, but not cell proliferation (25,26). This suggested that high concentrations of RAD001 or combination of RAD001 with other chemotherapeutic drugs is required for effective inhibition of cancer growth. Pretreatment with RAD001 was found to increase the sensitivity of CCA cells to oxaliplatin (27).

The mTOR pathway regulates multiple cellular signals, mediated by the mTORC1 and mTORC2 complexes. Previously, mTORC1 has been shown to regulate cellular processes, including cell proliferation, differentiation, cell cycle and protein translation; whereas, mTORC2 has been shown to regulate the actin cytoskeleton (5–7). RAD001, a derivative of rapamycin, has been considered to confer inhibition primarily via the inhibition of mTORC1 (28). However, the results of the present study demonstrated that RAD001 inhibits CCA cell invasion. This suggested that these events involve the activation of the ERK cascade, a central pathway that transmits signals from a number of extracellular agents to regulate cellular processes. The assertion is based on the observation that low doses of RAD001 suppress the phosphorylation of ERK1/2 and also inhibit the invasive property of CCA cells. These observations are consistent with our previous study showing that ERK1/2 activation is required for CCA cell invasion (2).

The results from the PathScan intracellular signaling array performed in the current study demonstrated that the level of phosphorylated S6 protein was decreased by RAD001 treatment. Ribosomal protein S6 is a component of a 40S ribosomal subunit which has been previously shown to be phosphorylated at Ser235/236 by S6K1, a downstream effector of mTOR (29). This phosphorylation is required for the translation of a group of mRNAs possessing a 5′-terminal oligopyrimidine tract. As predicted, S6 phosphorylation levels were decreased by RAD001 treatment, confirming that the mTOR signaling pathway is inhibited by RAD001.

In the current study, RAD001-treated cells also exhibited higher levels of AMPK phosphorylation compared with the control cells. AMPK is a highly conserved sensor of cellular energy status. In addition, a role for AMPK in the regulation of cancer cell invasion has recently been demonstrated and the pharmacological activation of AMPK reduces cancer cell invasion (30). Therefore, we suggested that the activation of AMPK by RAD001 inhibits the invasive property of CCA cells. Future studies must be performed to demonstrate the exact roles of AMPK in CCA cells.

BAD is a proapoptotic member of the Bcl-2 gene family and the proapoptotic activity of BAD is regulated through its phosphorylation. Only non-phosphorylated BAD induces apoptosis by forming a heterodimer with Bcl-2 and Bcl-xL, inactivating them and allowing Bax/Bak-triggered apoptosis (31). However, phosphorylation suppresses apoptotic activity due to sequestration of p-BAD in the cytoplasm by 14-3-3 proteins, preventing neutralization of the antiapoptotic BCL-2 proteins. Notably, the results from the PathScan intracellular signaling array kit performed in the current study showed that, under the condition where RAD001 induces apoptosis and reduces ERK1/2 phosphorylation, the level of p-Ser112 was significantly increased. This observation was unexpected since Ser112 is a known substrate of p90 ribosomal S6K (p90RSK), a downstream target of the mitogen-activated protein kinase pathway, thus, a reduction of ERK1/2 is predicted to parallel a reduction of the p-Ser112 of BAD. However, Ser112 is a substrate for multiple kinases in addition to p90RSK, including protein kinase A, PIM kinases and p21-activated kinases (32). It is possible that the reduction of p-Ser112 caused by the suppression of ERK1/2 activity is shadowed by an increase of phosphorylation by the other kinases. Although the present study showed that RAD001 treatment caused an enhancement of p-Ser112, apoptosis was induced. This may be explained by the evidence that the phosphorylation at Ser112 alone is not sufficient to suppress apoptosis. Previously, a tiered phosphorylation model was proposed, where the phosphorylation of at least two serine residues is required to fully neutralize the proapoptotic activity of BAD (33,34). In addition, the other proapoptotic proteins, including BH3 interacting-domain death agonist, Bcl-2 interacting protein, p53 upregulated modulator of apoptosis and NADPH oxidase activator 1 (35,36), may substitute for BAD in inducing apoptosis under this condition.

The results of the present study suggest a mechanism that controls CCA cell proliferation and invasion by activation of the mTOR signaling pathway. In addition, results showed that RAD001 may be a suitable new molecular target for CCA therapy.
